# Using geometric wing morphometrics to distinguish *Aedes japonicus japonicus* and *Aedes koreicus*

**DOI:** 10.1186/s13071-023-06038-y

**Published:** 2023-11-15

**Authors:** Felix G. Sauer, Wolf Peter Pfitzner, Hanna Jöst, Leif Rauhöft, Konstantin Kliemke, Unchana Lange, Anna Heitmann, Stephanie Jansen, Renke Lühken

**Affiliations:** 1 Bernhard Nocht Institute for Tropical Medicine, Hamburg, Germany; 2 Kommunale Aktionsgemeinschaft Zur Bekämpfung Der Schnakenplage e. V. (KABS), Georg-Peter-Süß-Str. 3, 67346 Speyer, Germany; 3 Faculty of Mathematics, Informatics and Natural Sciences, University of Hamburg, Hamburg, Germany

**Keywords:** *Aedes japonicus japonicus*, *Aedes koreicus*, Geometric wing morphometrics, Identification

## Abstract

**Background:**

*Aedes japonicus japonicus* (Theobald, 1901) and *Aedes koreicus* (Edwards, 1917) have rapidly spread in Europe over the last decades. Both species are very closely related and occur in sympatry. Females and males are difficult to distinguish. However, the accurate species discrimination is important as both species may differ in their vectorial capacity and spreading behaviour. In this study, we assessed the potential of geometric wing morphometrics as alternative to distinguish the two species.

**Methods:**

A total of 147 *Ae. j. japonicus* specimens (77 females and 70 males) and 124 *Ae. koreicus* specimens (67 females and 57 males) were collected in southwest Germany. The left wing of each specimen was removed, mounted and photographed. The coordinates of 18 landmarks on the vein crosses were digitalised by a single observer. The resulting two-dimensional dataset was used to analyse the differences in the wing size (i.e. centroid size) and wing shape between *Ae. j. japonicus* and *Ae. koreicus* using geometric morphometrics. To analyse the reproducibility of the analysis, the landmark collection was repeated for 20 specimens per sex and species by two additional observers.

**Results:**

The wing size in female *Ae. koreicus* was significantly greater than in *Ae. j. japonicus* but did not differ significantly for males. However, the strong overlap in wing size also for the females would not allow to discriminate the two species. In contrast, the wing shape clustering was species specific and a leave-one-out validation resulted in a reclassification accuracy of 96.5% for the females and 91.3% for the males. The data collected by different observers resulted in a similar accuracy, indicating a low observer bias for the landmark collection.

**Conclusions:**

Geometric wing morphometrics provide a reliable and robust tool to distinguish female and male specimens of *Ae. j. japonicus* and *Ae. koreicus*.

**Graphical Abstract:**

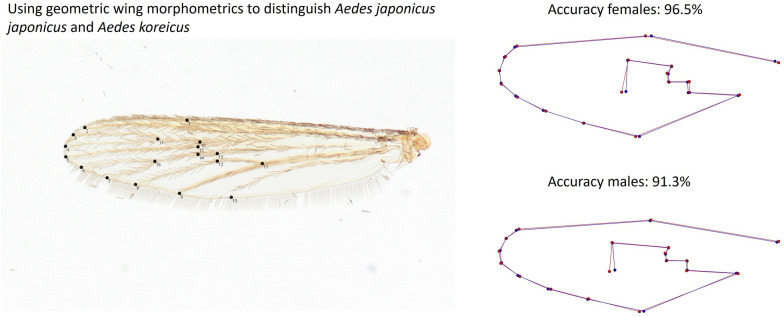

**Supplementary Information:**

The online version contains supplementary material available at 10.1186/s13071-023-06038-y.

## Background

The global spread of mosquito species poses a serious risk for public health, including nuisance and transmission of pathogens [1–3]. The most prominent representative in Europe is the Asian tiger mosquito (*Aedes albopictus* Skuse, 1984), which is an aggressive biter and potential vector of several pathogens. Establishment of the species in the Mediterranean region allowed the local circulation of chikungunya virus (CHIKV) and small outbreaks of dengue virus (DENV) and Zika virus (ZIKV) over the last 15 years [4–14].

Besides the Asian tiger mosquito, there are two further established exotic *Aedes* species in Europe: the Japanese bush mosquito (*Aedes japonicus japonicus*) and Korean bush mosquito (*Aedes koreicus*). The first established population of *Ae. j. japonicus* was detected in Belgium in 2002 [15]. In 2008, it was confirmed for Switzerland and bordering Germany [16], where it was later confirmed to be widespread [17]. Within 2 decades, the species is now present in wide parts of Germany [18]. The species is also considered to be established in Austria [19], Slovenia [20], The Netherlands [21], Hungary [22], France [23], Croatia [24], Bosnia-Herzegovina [25], Serbia [25], Italy [19], Liechtenstein [22] and Spain [26]. *Aedes koreicus* was first detected outside its native range in Belgium in 2008, where it established and overwintered, but did not seem to spread further [27, 28]. In contrast, the population detected in northeastern Italy in 2011 was observed to rapidly expand its distribution in Italy [29, 30] and towards Switzerland [31]. Established small populations have also been observed in Germany [32, 33], Hungary [34] and the north coast of the Black Sea in Ukraine and Russia [35].

Analysis of the host-feeding patterns demonstrated that *Ae. j. japonicus* and *Ae. koreicus* show a high prevalence of mammalian blood meals, including humans, while birds only play a minor role [36, 37]. The vector competence of both species is not well studied. For *Ae. j. japonicus*, experimental studies confirmed vector competence for several arboviruses, for example, Japanese encephalitis virus (JEV), West Nile virus, Saint Louis encephalitis virus, La Crosse virus and CHIKV [38–43]. The vector competence of *Ae. koreicus* is even less studied, but the species was experimentally proven to be a competent vector for *Dirofilaria immitis*, CHIKV and ZIKV [44–46]. Moreover, the species is considered to play a role as a vector of the JEV in Asia [47].

A reliable differentiation of exotic mosquito species is important to monitor the spread, initiate early control measurements or understanding the local risk of pathogen transmission. Eradication of exotic mosquito species was only demonstrated to be effective if the populations were detected in an early stage of establishment [48, 49]. From the established exotic mosquito species in Europe, *Ae. j. japonicus* and *Ae. koreicus* have the highest morphological similarity. The coloration of the hind femur, the pale basal scales on hind tarsomere IV and the subspiracular patch are considered distinctive characters to morphologically differentiate *Ae. koreicus* and *Ae. j. japonicus* [32, 50, 51]. However, these morphological differences are very subtle, and their accuracy for species identification varied between 91 and 100% depending on the respective studies [32, 50, 51]. In addition, the conditions of the samples can make morphological identification even for specialised entomologists difficult. Molecular assays for the differentiation of both species are well established with barcoding, e.g. the nad4 gene [52]. However, PCRs are still expensive, requiring specialised equipment and trained personal [53]. Geometric morphometric analysis of wings is an alternative low-cost method, proven to be suitable to analyse the evolution and population structure and for the correct species identification of mosquitoes. The method showed similar accuracy to molecular barcoding [54] and is even suitable to differentiate cryptic mosquito species, e.g. *Culex pipiens pipiens* biotype *pipiens* Linnaeus, 1758 (*Cx. p. pipiens*), and *Cx. torrentium* Martini, 1925 [55]. Martinet et al. [56] successfully used wing morphometry to differentiate *Ae. j. japonicus* and *Ae. albopictus*. However, their study did not include *Ae. koreicus* and was only focused on males. Thus, the aim of the present study was to evaluate whether geometric wing morphometrics represents a reliable tool to differentiate *Ae. koreicus* and *Ae. j. japonicus* female and male specimens.

## Methods

### Field work and rearing

Ovitraps were used to collect eggs of *Ae. j. japonicus* and *Ae. koreicus* from different sites in the southwestern region of Germany (see Table 1 for the coordinates of the sampling sites) during the summer of 2021, following the field method described by Pfitzner et al. [32]. The sticks with eggs were submerged in water for 3 days, air-dried for 2 days and then submerged again for 3 days to allow the hatching of delayed eggs that had not hatched during initial immersion. The mosquitoes were reared in a controlled environment with a temperature of 26 °C, a relative humidity of 70% and a light:dark cycle of 12:12 h, including a 30-min twilight period. Larvae were fed every 2–3 days with Catfish Chips Nature (Sera, Heinsberg, Germany). Mosquitoes were fed ad libitum using cotton pads soaked with an 8% fructose solution (Carl Roth, Karlsruhe, Germany), which were refreshed every 2–3 days.

**Table 1:** Overview of the number of mosquitoes per sampling site

Sampling site	Longitude	Latitude	*Aedes j. japonicus*		*Aedes koreicus*	
			Female	Male	Female	Male
Budenheim	8.167114	50.024837	0	1	0	0
Rheinau Freistett	7.934067	48.685016	18	5	0	0
Weinheim	8.672	49.524	59	52	0	0
Wiesbaden Mainz-Kastel	8.281782	50.015663	0	0	1	4
Wiesbaden Naurod	8.298918	50.134383	0	2	14	10
Wiesbaden Südfriedhof	8.268892	50.05853	0	10	52	43

### Molecular identification

Identification of all specimens was confirmed by DNA barcoding. DNA isolation was performed from one leg per specimen adapting the protocol described by Blattner et al. [57]. Individual legs were placed into 2-ml tubes, and 180 µl ATL buffer (Qiagen) and 20 µl (20 mg/ml) Proteinase K (Qiagen) were added followed by incubation overnight at 56 °C on a shaking thermomixer (400 rpm). Extraction with the DNeasy Blood & Tissue kit (Qiagen) was performed according to the manufacturer’s protocol. Polymerase chain reaction (PCR) amplification of the COI (*Ae. j. japonicus*) and nad4 gene region (*Ae. koreicus*) was conducted with the protocol published by Fang et al. [58] and Fonseca et al. [52]. All amplicons were further processed with Sanger sequencing (LGC Genomics, Berlin, Germany), pre-processed with Geneious 7.1.9 (https://www.geneious.com) and compared to GenBank sequences (http://blast.ncbi.nlm.nih.gov/Blast.cgi). Representative sequences for both species have been submitted to GenBank (accession no. OR699056, OR699057, OR723972, OR723973, OR723974).

### Wing preparation and landmark collection

In total, 147 *Ae. j. japonicus* specimens (77 females and 70 males) and 124 *Ae. koreicus* specimens (67 females and 57 males) were included in the study. The left wing of each mosquito was removed and mounted in Euparal (Carl Roth, Karlsruhe, Germany) on microscopic slides and dried. Subsequently, the mounted wings were photographed (Olympus DP23, Olympus GmbH, Tokyo, Japan) under 20 × magnification with a stereomicroscope (Olympus SZ61, Olympus GmbH, Tokyo, Japan). The collection of landmark coordinates for 18 wing vein crosses was performed with the multi-point tool in Fiji [59] as bioscience bundle of imageJ [60]. The selected landmarks are consistent with a variety of studies analysing the interspecific wing shape variation of mosquitoes, e.g [54, 61–63]. All landmark coordinates were collected by a single observer (author KK). To assess the degree of observer bias in the landmark collection, the measurements were repeated for 20 randomly selected images per species and sex by two observers (authors FGS and LR).

### Statistics

The two-dimensional landmark coordinates were used to calculate the centroid size and the superimposed shape coordinates of each specimen with the “gpagen” function in the R package “geomorph”, version 4.0.1 [64]. The centroid size is considered a proxy for wing size and was used to statistically compare the mean wing size of *Ae. j. japonicus* and *Ae. koreicus* through an analysis of variance (ANOVA). As mosquitoes are well known for sex-specific differences in their wing size [65], ANOVA was applied separately for the females and males. The allometric effects of the centroid size on the wing shape were assessed with the “procD.lm” function using 1000 permutations [64]. The variability in the superimposed wing shape coordinates between the specimens was visualised by principal component analyses (PCA). In addition, the wing shape coordinates were analysed by linear discriminant analyses (LDA) with the R package “MASS”, version 7.3.58.2 [66] to classify *Ae. j. japonicus* and *Ae. koreicus*. Subsequently, the obtained species classification from the LDA was cross-validated (leave-one-out method) to test the classification accuracy. The LDA and cross-validation were also done separately for both sexes since mosquitoes have sex-specific wing shape differences and should not be mixed in the same analyses when researchers are interested in species-specific differences [67]. The mean shape configuration of the 18 landmarks was calculated to visualise differences between female and male *Ae. j. japonicus* and *Ae. koreicus*. In addition, the superimposed shape coordinates of each landmark were plotted individually to visually inspect their importance for species identification.

A potential observer effect on the centroid size was assessed with an ANOVA by means of the “prcoD.lm” function in gemorph using 500 permutations [67]. Two ANOVAs were conducted for the wing size of females and males with the three observers as categorial covariate. The effect of different observers on the wing shape coordinates was assessed through the “morphol.disparity” function in gemorph using 500 [67]. Thereby, the morphological disparity, i.e. mean Procrustes variance, was calculated for the three measurements per wing and for the two species. This was also done separately for males and females. Based the resulting morphological disparity only, it is difficult to interpret the influence of disparity due to different observers on the actual species classification accuracy. Therefore, we conducted an additional LDA and reclassified the results with a leave-one-out cross-validation for all specimens, which were measured by three different observers. These analyses involved a dataset with replicated measurements of individual wings and should therefore not be interpreted as final accuracy. Instead, it was conducted to analyse the effect of different observers on the species classification accuracy to get deeper insight into the robustness of geometric morphometrics to differentiate the target species. All statisitical analyses and visulisation were conducted in R, version 4.2.3 [68], including the package ggplot2, version 3.4.0 [69].

## Results

**Fig. 1:**
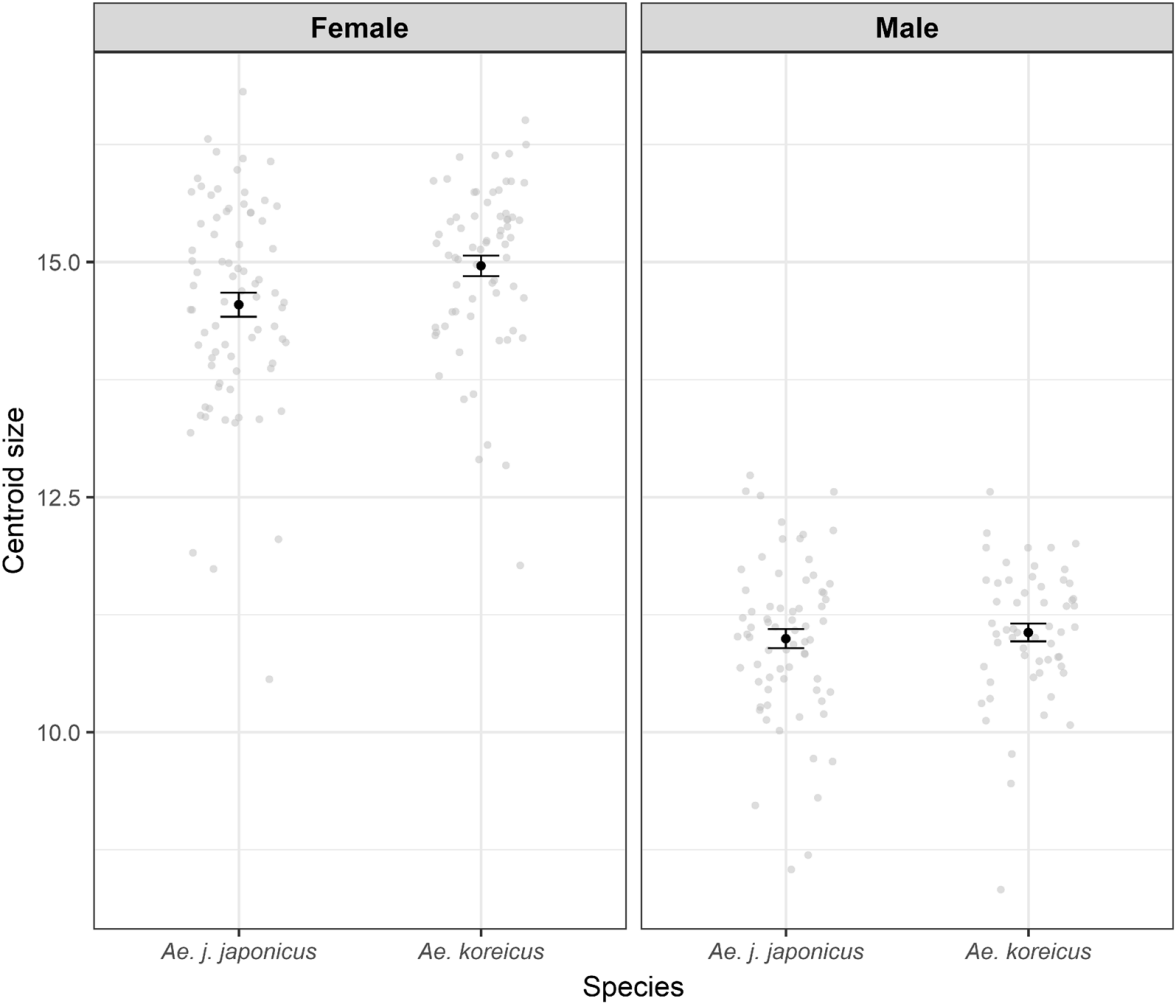
Centroid size of *Aedes japonicus japonicus* and *Aedes koreicus* for female and male specimens. Grey dots represent the centroid size of one specimen. The black dots mark the mean centroid size with 95% confidence interval as error bars

**Fig. 2:**
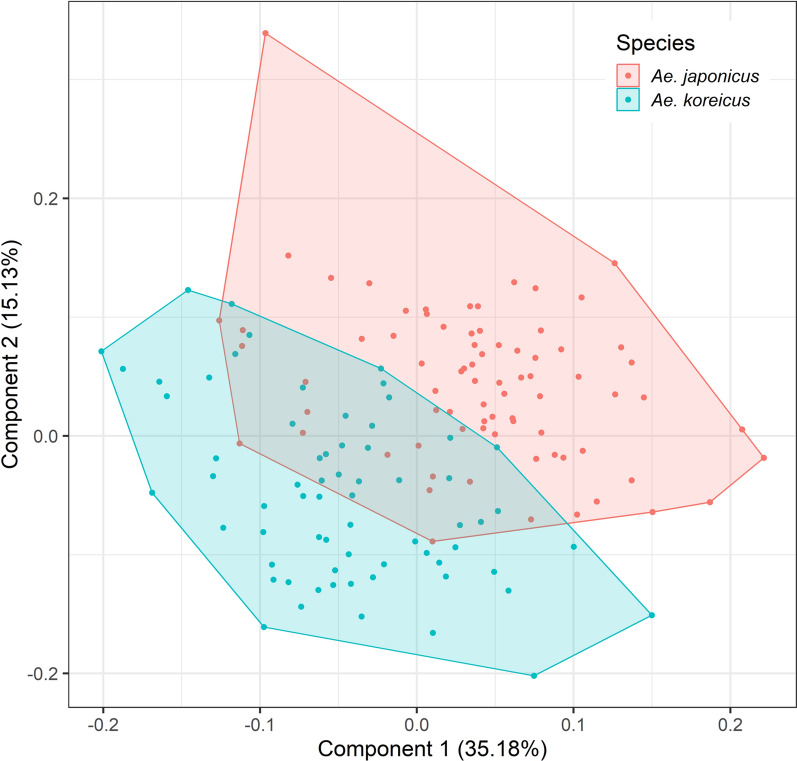
Principal component analysis of the wing shape variation of female *Aedes japonicus japonicus* and *Aedes koreicus*

**Fig. 3:**
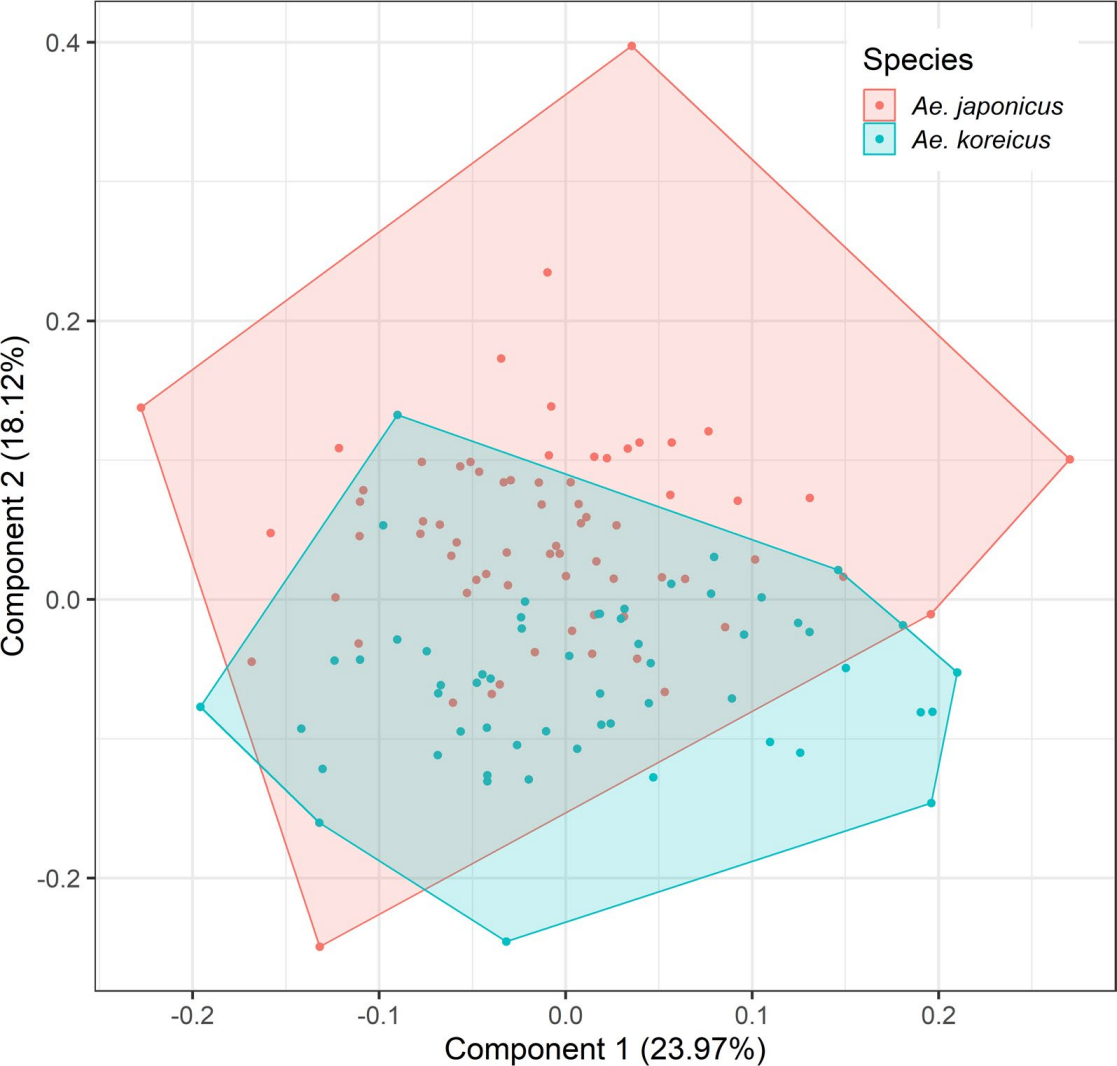
Principal component analysis of the wing shape variation of male *Aedes japonicus japonicus* and *Aedes koreicus*

The mean centroid size of the female *Ae. koreicus* specimens was significantly greater than for female *Ae. j. japonicus* specimens (*F*_1,142_ = 5.82, *P* = 0.017), but no significant difference was observed between the males of both species (*F*_1,125_ = 0.22, *P* = 0.641) (Fig. 1). A low but statistically significant allometric effect on the wing shape could be registered in males (*F*_1,125_ = 5.55, *R*^2^ = 0.043, *P* < 0.001) and females (*F*_1,142_ = 3.9, *R*^2^ = 0.027, *P* = 0.006). The first two principal components of the PCAs explained 50.3% of the wing shape variation in the females (Fig. 2) and 42.1% of the wing shape variation in the males (Fig. 3). In both sexes, an overlap between the two species-specific clusters was observed (Figs. 2, 3).

**Fig. 4:**
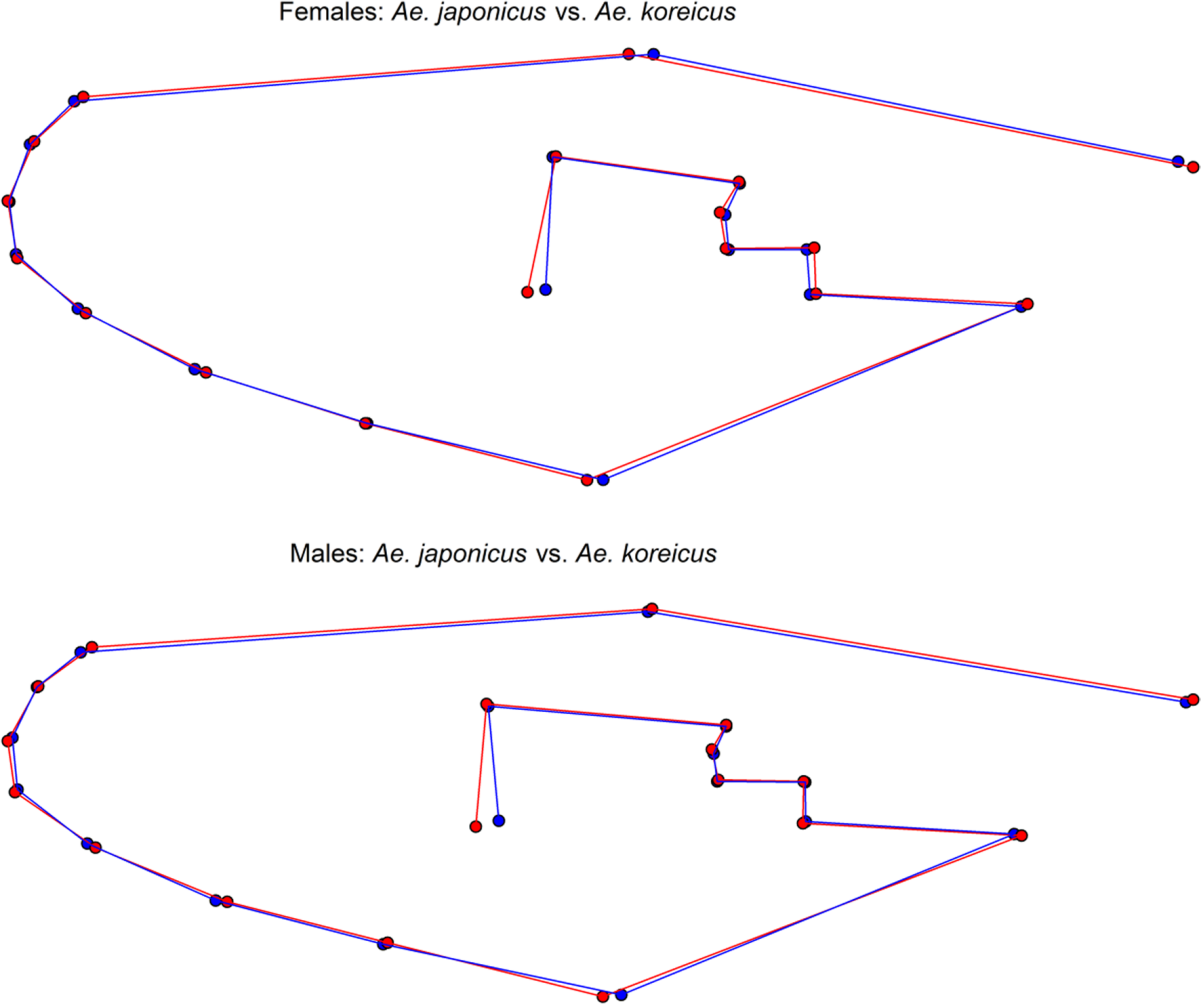
Comparison of the mean shape variation of the superimposed landmarks between *Aedes japonicus japonicus* (red) and *Aedes koreicus* (blue)

The leave-one-out cross-validation based on the classification of the LDA revealed a total accuracy of 96.5% for the species identification of the females. Three of 77 female *Ae. j. japonicus* specimens were misclassified as *Ae. koreicus* and two of 67 female *Ae. koreicus* specimens were misclassified as *Ae. j. japonicus*. The species identification accuracy (leave-one-out method) for the males was 91.3%. Thereby, seven of 70 *Ae. j. japonicus* specimens and four of 57 *Ae. koreicus* specimens were falsely classified. The highest mean shape variation between both species and both sexes was observed for landmark 18 (Fig. 4). However, the variation of all superimposed shape coordinates, including landmark 18, showed a strong overlap between both species, indicating that none of the landmarks alone provide enough information for an accurate species identification (Additional file 1: Figs. S1 and S2).

### Observer effect

The centroid size did not significantly differ among the three observers for both, females (linear model, *F* = 0.276, *Z* = − 0.751, *R*^2^ = 0.005, *P* = 0.776) and males (linear model, *F* = 0.219, *Z* = − 0.848, *R*^2^ = 0.004, *P* = 0.784). The mean Procrustes variance for the repeated measurements per specimen was 0.000439 for the females and 0.00034 for the males and thereby considerably smaller than the mean Procrustes variance observed within the two species, *Ae. j. japonicus* (females: 0.001686, males: 0.001494) and *Ae. koreicus* (females: 0.00094, males: 0.001639). This indicates a relatively low observer effect and was also confirmed by the leave-one-out classification with the wing data set of the repeated measurements, which yielded a similar accuracy as the dataset produced by a single observer for the females (95.8%) and an even a higher accuracy for the males (97.5%).

## Discussion

*Aedes. japonicus japonicus* and *Ae. koreicus* are invasive mosquitoes in Europe, which are established in different countries [15–19, 21–35]. Analysis of the vector competence [38–46] and host-feeding patterns [36, 37] of the two species indicate a vector capacity of a variety of pathogens for both, e.g. ZIKV and CHIKV. However, monitoring of the spread of both sympatric occurring species is difficult because of their morphological similarity.

The wing size was statistically significantly different between the females but not for the males of both species. On average, female *Ae. koreicus* showed a larger centroid size than *Ae. j. japonicus*. However, although statistically significant, the wing size showed a very strong overlap and is not a reliable feature to discriminate between *Ae. j. japonicus* and *Ae. koreicus* females. This is a common phenomenon, which was demonstrated for several taxonomic groups including mosquitoes [70–72]. The wing size is strongly affected by local environmental factors, e.g. temperature or food availability in the breeding sites [73], resulting in a high intraspecific wing size variability which does not allow a clear species identification based on size, only.

However, it has again been shown that the geometric morphometric analysis of the wing shape is a powerful tool for the identification of mosquitoes. Its use is of particular interest when molecular identification cannot be performed or when damaged mosquitoes hinder an accurate morphological identification. Our study provides the first morphometric information about *Ae. koreicus*. We demonstrated that *Ae. j. japonicus* and *Ae. koreicus* can be identified with a high classification accuracy (96.5% for females, 91.3% for males), which can otherwise morphologically only be distinguished by very subtle differences [51]. In both sexes, the strongest difference between *Ae. j. japonicus* and *Ae. koreicus* was observed for landmark 18, i.e. where the media bifurcates into *M*_1+2_ and *M*_3+4_. However, none of the landmarks alone showed enough divergence to clearly distinguish *Ae. j. japonicus* and *Ae. koreicus.* Hence, the full set of landmarks and geometric morphometric analysis is required to differentiate the two species.

We analysed the repeatability of landmark collection by three different observers with a subset of the mosquito wings. The results demonstrated that the observer bias plays no or only a minor role when studying in the centroid size of mosquito wings. However, an observer effect should be considered in the shape analysis. Thereby, the observer bias was lower for males than for females. Unlike most other Dipteran families, mosquitoes have scales on their wing veins, which can obstruct a clear view on the vein crosses. For *Ae. j. japonicus* and *Ae. koreicus*, these scales are less dense in males compared to females, i.e. the vein crosses are more easily visible. This likely increases the reproducibility of landmark collection for males. As demonstrated by Lorenz and Suesdek [74], the removal of the wing scales can improve the accuracy and reproducibility in landmark-based geometric morphometrics but also increases the effort for wing preparation. In our study, we did not remove wing scales, but still obtained a high accuracy to classify the two species, even when the landmarks were collected by different observers. This underpins the robustness of geometric wing morphometrics to distinguish *Ae. j. japonicus* and *Ae. koreicus*. Nevertheless, when interested in more subtle differences in the wing shape, e.g. intraspecific patterns, the removal of the wing scales and a single observer should still be considered to increase the accuracy of the landmark coordinates.

## Conclusions

As previously demonstrated for the separation between *Ae. j. japonicus* and *Ae. albopictus* [56], *Cx. p. pipiens* and *Cx. torrentium* [55] or *Anopheles* species [75], our study again demonstrated that geometric wing morphometrics is a powerful tool for the identification of mosquito species. Future research should especially focus on the development of user-friendly tools for a quick landmark collection and subsequent species identification, e.g. using deep learning methods for automatic landmark detection [76].

### Supplementary Information

**Additional file 1. Figure S1**: Variation of the superimposed shape coordinates for each landmark of the female specimens. **Figure S2**: Variation of the superimposed shape coordinates for each landmark of the male specimens.

## Data Availability

All data generated by this study and used is presented within this published article and supplementary files. All wing images are stored and available at: 10.5061/dryad.zcrjdfnjn.
