# Supplementary material for: Using geometric wing morphometrics to distinguish Aedes japonicus japonicus and Aedes koreicus
Source: Parasit Vectors. 2023 Nov 15;16:418. doi: 10.1186/s13071-023-06038-y (PMC10648383; doi:10.1186/s13071-023-06038-y)
Supplement: Supplementary file 1 — Additional file 1. Figure S1: Variation of the superimposed shape coordinates for each landmark of the female specimens. Figure S2: Variation of the superimposed shape coordinates for each landmark of the male specimens. [file 13071_2023_6038_MOESM1_ESM.docx]

**Using geometric wing morphometrics to distinguish *Aedes japonicus japonicus* and *Aedes koreicus***

Felix G. Sauer^1*^, Wolf Peter Pfitzner^2^, Hanna Jöst^1^, Leif Rauhöft^1^, Konstantin Kliemke^1^, Unchana Lange^1^, Anna Heitmann^1^, Stephanie Jansen^1,3^, Renke Lühken^1^

**Affiliations**

^1^Bernhard Nocht Institute for Tropical Medicine, Hamburg, Germany

^2^Kommunale Aktionsgemeinschaft zur Bekämpfung der Schnakenplage e. V. (KABS), Georg-Peter-Süß-Str. 3, 67346, Speyer, Germany

^3^Faculty of Mathematics, Informatics and Natural Sciences, University of Hamburg, Hamburg, Germany

**Supplementary Information**

The plots visualise the variation between *Aedes japonicus japonicus* and *Aedes koreicus* in the superimposed shape coordinates for each landmark.

**Figure 1: Variation of the superimposed shape coordinates for each landmark of the female specimens**









**Figure 2: Variation of the superimposed shape coordinates for each landmark of the male specimens**

**

**

**

**

**

**
